# Prevalence and associated factors of double and triple burden of malnutrition among child-mother pairs in Ethiopia: Spatial and survey regression analysis

**DOI:** 10.1186/s40795-022-00528-5

**Published:** 2022-04-21

**Authors:** Bethelihem Tigabu Tarekegn, Nega Tezera Assimamaw, Kendalem Asmare Atalell, Selam Fisiha Kassa, Addis Bilal Muhye, Masresha Asmare Techane, Tewodros Getaneh Alemu, Chalachew Adugna Wubneh, Getaneh Mulualem Belay, Tadesse Tarik Tamir, Destaye Guadie Kassie, Amare Wondim, Bewuketu Terefe, Mohammed Seid Ali, Beletech Fentie, Almaz Tefera Gonete, Berhan Tekeba, Bogale Kassahun Desta, Melkamu Tilahun Dessie, Amare Demsie Ayele

**Affiliations:** 1 Department of Pediatrics and Child Health Nursing, School of Nursing, College of Medicine and Health Sciences, University of Gondar, Gondar, Ethiopia; 2 School of Nursing, College of Medicine and Health Sciences, University of Gondar, Gondar, Ethiopia

**Keywords:** Double Burden, Triple Burden, Malnutrition, Under Five Children, Spatial Distribution, Hotspot, Survey Regression, Associated Factors, Ethiopia

## Abstract

**Background:**

Evidence on double and triple burdens of malnutrition at household level among child-mother pairs is a key towards addressing the problem of malnutrition. In Ethiopia, studies on double and triple burdens of malnutrition are scarce. Even though there is a study on double burden of malnutrition at national level in Ethiopia, it doesn’t assess the triple burdens at all and a few forms of double burden of malnutrition. Therefore, this study aimed to determine the prevalence and associated factors of double and triple burdens of malnutrition among child-mother pairs in Ethiopia.

**Methods:**

A total sample of 7,624 child-mother pairs from Ethiopian Demographic and Health Survey (EDHS) 2016 were included in the study. All analysis were performed considering complex sampling design. Anthropometric measures and hemoglobin levels of children, as well as anthropometric measurements of their mothers, were used to calculate double burden of malnutrition (DBM) and triple burden of malnutrition (TBM). Spatial analysis was applied to detect geographic variation of prevalence of double and triple burdens of malnutrition among EDHS 2016 clusters. Bivariable and multivariable binary survey logistic regression models were used to assess the factors associated with DBM and TBM.

**Results:**

The overall weighted prevalence of DBM and TBM respectively were 1.8% (95%CI: 1.38–2.24) and 1.2% (95%CI: 0.83–1.57) among child-mother pairs in Ethiopia. Significant clusters of high prevalence of DBM and TBM were identified. In the adjusted multivariable binary survey logistic regression models, middle household economic status [AOR = 0.23, 95%CI: 0.06, 0.89] as compared to the poor, average birth weight [AOR = 0.26, 95%CI: 0.09, 0.80] as compared to large birth weight and children aged 24–35 months [AOR = 0.19, 95%CI: 0.04,0.95] as compared to 6–12 months were less likely to experience DBM. Average birth weight [AOR = 0.20, 95%CI: 0.05, 0.91] as compared to large birth weight and time to water source <=30 min [AOR = 0.41, 95%CI: 0.19,0.89] as compared to on premise were less likely to experience TBM.

**Conclusion:**

There is low prevalence of DBM and TBM among child-mother pairs in Ethiopia. Interventions tailored on geographic areas, wealth index, birth weight and child birth could help to control the emerging DBM and TBM at household level among child-mother pairs in Ethiopia.

## Background

Apart from having enough food, having access to varied sources of food can help ensure diet quality, so that diets are sufficient not only in calories but also in micronutrients [1]. An imbalance in the amount of calories, protein and/or other nutrients consumed is referred to as malnutrition which commonly encompasses either undernutrition, overnutrition or micronutrient deficiencies. Undernutrition is one form of malnutrition that commonly include being underweight, stunted, and wasted. It is critical to prevent undernutrition and micronutrient deficiencies since they are risk factors for child death, poor development, and early adult mortality [2, 3]. Moreover, overweight or obesity in women is positively associated with several adverse maternal and fetal consequences throughout the period of pregnancy, delivery and the postpartum [4, 5].

Malnutrition, in its many forms, has previously been understood and approached as a separate public health concern. However, much less is known about the simultaneous occurrence of undernutrition and overnutrition in developing countries, which is the new emergent reality that undernutrition and overnutrition are interconnected. As a result, for policy solutions to be effective, double-duty acts that address more than one dimension must be undertaken. Hence, understanding the double burden of malnutrition (DBM) and triple burden of malnutrition (TBM) might be critical to accomplishing the goal of eradicating childhood malnutrition.

The DBM is referred to as the coexistence of undernutrition and overnutrition in the same nations, communities, or families [6]. The co-existence of an overweight or obese mother with undernourished child in the same household is an essential issue [7, 8]. In this study, the DBM includes undernourished child (underweight, stunted or wasted), and maternal overnutrition (overweight or obese for one’s height). There are many countries worldwide facing DBM [9]. The TBM, on the other hand, refers to the co-existence of micronutrient deficiencies, undernutrition and overnutrition [10, 11]. Micronutrient deficiencies include inadequate consumption of vitamins and minerals. In this study, the TBM includes undernourished child (underweight, stunted or wasted), child micronutrient deficiency (anemia, which is often due to iron deficiency), and maternal overnutrition (overweight or obese for one’s height). It is one of the leading causes of disease worldwide, affecting every country, particularly low- and middle-income countries.

Globally, child undernutrition is widespread and continues to be a major concern. Accordingly, an estimated 22.0%, and 6.7% of children under five worldwide were stunted and wasted respectively [12]. Of which, most children with malnutrition live in Africa and Asia [12]. On the other hand, according to a recent review report in low- and middle income countries, 29.1% of children under the age of five years are stunted, 13.7% underweight, and 6.3% wasted in 2006–2018 [13]. Furthermore, undernutrition is responsible for roughly half of all fatalities among children under five. An estimated 6.3 million live-born children worldwide perished before the age of five due to undernutrition [14].

Despite the fact that Ethiopia continues to suffer with the burden of malnutrition, problems associated with DBM and/ or TBM are emerging as a public health concerns. When DBM is prevalent, it cannot be addressed only by overnutrition policies [15]. Maternal and child malnutrition in low-and-middle-income countries (LMIC) includes both undernutrition and the rapidly developing issues linked with overweight and obesity [16]. Inadequate intake of essential nutrients may weaken immune systems, hinder brain development, and increase the risk of conditions including anemia [17]. In this context, two billion individuals worldwide are affected by iron deficiency anemia.

Despite the fact that a number of public health interventions have been adopted to address the various forms of malnutrition in Ethiopia, the DBM and TBM yet represent a new and major setback for Ethiopian nutrition policy. Ethiopia has made progress in eliminating hunger and, to a lesser extent, undernutrition; yet, malnutrition continues to be one of Ethiopia’s primary public health challenges. As a consequence; the government adopted a National Nutrition Program, constructed an infant and young child feeding manual, and established a monthly child growth and monitoring program. However, it remains to be a serious public health issue in Ethiopia. The majority of research on child malnutrition in Ethiopia are descriptive, with just a few being analytical, and depending on pocket area survey data that may be difficult to generalize across varied Ethiopia. Even while there is a nationwide research study on the DBM in Ethiopia [18], it has limitations. It doesn’t assess the TBM at all and some forms of DBM among child-mother pairs.

As a result, the purpose of this study is to determine the prevalence and associated factors of the double and triple burdens of malnutrition among child-mother pairs in Ethiopia. The evidence from this study will assist policy makers, program designers and implementers in taking appropriate action to achieve sustainable development goal (SDG) 2030, specifically eradicating all forms of malnutrition in Ethiopia by 2030.

## Methods

### Study setting

This study is a further analysis of nationwide data, Ethiopian Demographic and Health Survey (EDHS) data, which was collected between January 18, 2016 and June 27, 2016 [19]. The 2016 EDHS is the fourth national representative cross-sectional survey to be conducted as part of the global MEASURE DHS initiative, which is led by the Ethiopian Central Statistical Agency (CSA). The target population for this study was pairs of all 6–59 month-old children with their mothers or caregivers in Ethiopia. Furthermore, the study population was pairs of all 6–59 month-old children with their respective mothers or caregivers in the randomly selected enumeration areas (EAs) of Ethiopia.

### Sampling procedures

**Fig. 1:**
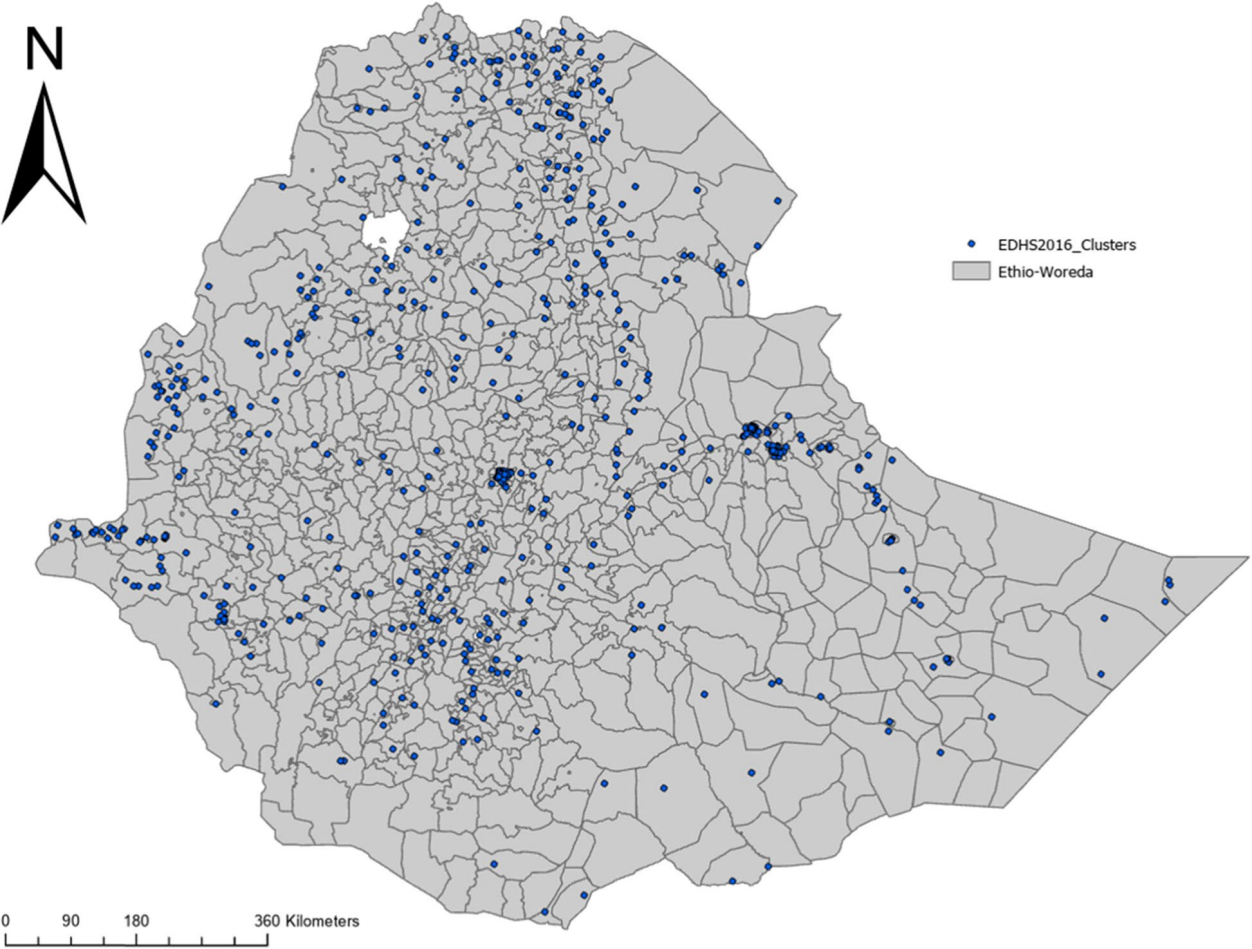
Clusters included Ethiopian DHS-2016

Briefly, the 2016 EDHS employed stratified, two-stage cluster sampling to identify the representative samples. The sampling frame for the 2016 EDHS consists of a total of 84,915 Enumeration Areas (EAs). In the first stage, 645 (202 urban and 443 rural) EAs were chosen. Figure 1 presents the map of survey cluster (EA) locations where raw dataset were collected. Then, in the second stage, a fixed number of 28 households was chosen from each enumeration area. A total of 16,650 households were included in the survey. A nationally representative population of 9,504 children aged 6–59 months in the chosen households and a total of 15,683 women aged 15—49 years were interviewed with a 95% response rate. A thorough description of the survey design and sampling procedure can be found elsewhere [19].

The unit of analysis was the child-mother/caregiver pair. The primary and secondary sampling units were clusters and households, respectively. To account for non-response, post-stratified weights were considered [20, 21]. For selected survey years, sociodemographic and anthropometric data from children under the age of five and their mothers (aged 15–49 years) were retrieved. Hence, in this study, 7,624 under-five child-mother/caregiver pairs with complete anthropometric and hemoglobin records were included.

### Variables of the study

In this study, we looked at two binary outcome variables. Specifically, the double burden of malnutrition (DBM) and triple burden of malnutrition (TBM) among child- to- mother/caregiver pairs.

DBM among child – mother/caregiver- pairs was defined based on related literatures [22–25]. That is, Yi = 1 when a child is undernourished (either stunted, wasted, or under-weight based on the World Health Organization (WHO) child growth standards [26]) and the mother/caregiver is over-nourished (overweight/obese), and Yi = 0 when neither is the case. Using the WHO standards of BMI (weight (kg)/height (m2)) [27], maternal nutritional status was classified as underweight (≤ 18.4); normal (18.5–24.9); overweight (25.0–29.9); and obese (≥ 30 kg/m2).

Based on related studies, the TBM was defined as a combination of the DBM of a child-mother pair plus an anemic child [22]. TBM includes undernourished children (either underweight, stunted, or wasted), an anemic child (with a micronutrient deficit, which is frequently caused by iron deficiency), and an over-nourished mother/caregiver (with a weight higher than healthy for height, either overweight or obese).

The double and triple burden of malnutrition among child-mother pairs in Ethiopia is the result of a number of complicated, multifarious, and interconnected variables that work at several levels, one of which is maternal-related characteristics. Accordingly, the independent variables were selected based on literature and their availability in the data used. Household (HH), mother and child characteristics are among the independent variables considered in this study. Specifically, residence (urban or rural) [22, 25, 28], child age [22, 25, 28], HH wealth [22, 23, 25, 28], mother’s age [22, 28], mother’s education level [22, 23, 28], and child sex [22, 28]. Other socio-demographic and environmental factors include employment status, marital status, family size, source of drinking water, and type of toilet facility [22].

### Data processing and analysis

The EDHS 2016 survey data sets and the Global Positioning System (GPS) points were obtained and processed with permission from Measure DHS (http://www.dhsprogram.com). The variables were then retrieved from the survey data for the children’s data sets.

For spatial analysis, ArcGIS Pro version 2.4 was utilized, while R was used for the remaining analyses. Because of the nature of the sampling design, all analyses were performed utilizing the complex sampling design adjustment approach and non-response rate. A survey package of R [29] was used to estimate confidence intervals around prevalence by taking sample weights into account, which represent the inverse of the chance that the observation is included.

To estimate the prevalence of DBM and TBM across socio-demographic determinant variables, descriptive statistics were utilized. The prevalence of overweight/obese mother and stunted child (OM/SC), overweight/obese mother and wasted child (OM/WC), overweight/obese mother and underweight child (OM/UC), overweight/obese mother and anemic child (OM/AC), DBM, and TBM were presented as weighted percentages with 95% confidence intervals (CIs).

Further, the GPS coordinates were then combined with the prevalence of DBM and TBM in each of the EDHS 2016 clusters. As a result, the cluster level prevalence of DBM and TBM was exported into ArcGIS to depict hot and cold spots of clusters. Geographic variation in DBM and TBM prevalence among EDHS clusters was identified using spatial analysis [30, 31]. Geographic variation of significant high prevalence or low prevalence of DBM and TBM was computed for each cluster using the Moran’s I statistic [30]. Maps depicting the distribution and variations of DBM and TBM throughout the country were constructed. In addition, as a complement to Moran’s I statistic, inverse distance weighted interpolation [31] was employed to estimate these distributions.

The standard binary logistic regression estimates are inadequate for a data from a complex survey design since the data originates from a complex survey design with stratification, clustering, and unequal weighting [32]. If the complex survey design is not included in the analysis, the standard errors are likely be underestimated, perhaps leading to statistically significant findings when they are not [20, 21, 32]. As a result, the survey binary logistic regression model [32] was used to analyze data in order to account for the complex sampling design. The bivariate and multivariable survey binary logistic regression models were used to evaluate the associated factors of the DBM and TBM. Bivariate analysis was used to examine the relationship between socio-demographic characteristics and outcome variables. The multivariable analysis included all variables with p-value less than or equal to 0.25 in the bi-variable analysis. Variables with a p-value of < 0.05 were considered statistically significant in multivariable analysis.

To demonstrate the strength of the association, the adjusted odds ratio (AOR) with the accompanying 95% CIs were provided.

## Results

### Characteristics of study participants

We included 7,624 pairs of children aged 6–59 months and their mothers, with anthropometric and hemoglobin measurements. Weighted mean age of children was 32 months (SD = 15.75). Weighted median age of the mother/caregivers was 29 years (IQR: 25–35). Weighted median household size was 6 (IQR: 4–7). Weighted median time to source of drinking water was 30 min (IQR: 10–60).

### Prevalence of malnutrition

The weighted prevalence of child and maternal indicators are given (Table 1). The burden of anemia among children were major public health problem (57.4%, 95%CI: 54.8, 59.8) followed by stunting (40.9%, 95%CI: 38.78, 42.95) in Ethiopia by 2016.

**Table 1:** Prevalence of nutritional status of children and mothers/caregivers (*n* = 7,624)

Indicator	Weighted Percent [95% CI]
Child Stunting	40.9 [38.78, 42.95]
Child Wasting	25.4 [23.78, 27.08]
Child underweight	9.4 [8.27, 10.51]
Child anemia	57.4 [54.8, 59.8]
Maternal Overweight/Obesity	5.8 [4.94, 6.59]

## Prevalence of double and triple burdens of malnutrition

Specifically, 3.1% (95%CI: 2.61–3.69) and 1.6% (95%CI: 1.18–1.99) of child-mother pairs experienced co-existence of overweight/obese mother and anemic child (OM/AC), and overweight/obese mother and stunted child (OM/SC) respectively (Table 2).

**Table 2:** The coexistence of various forms of malnutrition (*n* = 7,624)

Co-existence of malnutrition	Weighted Percent [95%CI]
OM/AC	3.1 [2.61, 3.69]
OM/SC	1.6 [1.18,1.99]
OM/WC	0.8 [0.49, 1.04]
OM/UC	0.3 [0.07, 0.45]

The overall weighted prevalence of DBM and TBM respectively were 1.8% (95%CI: 1.38–2.24) and 1.2% (95%CI: 0.83–1.57) in Ethiopia (Table 3). The detailed prevalence of the indicators by factors are also given. In the same table, DBM and TBM by selected characteristics are given. Accordingly, prevalence of DBM among urban residents (3.6%, 95%CI: 2.40–4.88) were higher than among rural residents (1.6%, 95%CI:1.14–2.06). Furthermore, based on maternal age the highest prevalence of DBM (2.7%, 95%CI:1.45–4.03) was observed among those in age range of 30–34 years old while the smallest prevalence (1.1%, 95%CI:0.29–1.83) in age group of 15–24 years old.

**Table 3:** Weighted prevalence of DBM and TBM by socio-demographic characteristics of the study participants

Variables		DBM [95%CI]	TBM [95%CI]
Toilet facility	Un Improved	1.9 [1.27, 2.46]	1.1 [0.62, 1.53]
	Improved	2.9 [1.81, 3.98]	1.8 [0.97, 2.71]
	Open Field	1.5 [0.70, 2.22]	1.2 [0.48, 1.96]
Mother’s Age	15–24	1.1 [0.29, 1.83]	0.9 [0.16, 1.55]
	25–29	1.5 [0.87, 2.17]	1.1 [0.52, 1.63]
	30–34	2.7 [1.45, 4.03]	1.8 [0.69, 2.86]
	35–49	1.9 [1.10, 2.73]	1.1 [0.44, 1.77]
HH size	2–4	1.6 [0.65, 2.50]	1.3 [0.42, 2.24]
	5–7	1.8 [1.16, 2.39]	1.1 [0.57, 1.54]
	8 +	2.1 [0.95, 3.29]	1.4 [0.42, 2.31]
Residence	Urban	3.6 [2.40, 4.88]	1.6 [0.76, 2.39]
	Rural	1.6 [1.14, 2.06]	1.2 [0.76, 1.56]
Education Mother’s	No Formal Education	1.8 [1.24, 2.41]	1.2 [0.76, 1.73]
	Primary	1.8 [1.01, 2.60]	1.2 [0.47, 1.93]
	Secondary/Higher	1.7 [0.55, 2.79]	0.7 [0.21, 1.16]
Time to water source (in minutes)	On Premise	2.8 [1.77, 3.85]	1.7 [0.80, 2.61]
	< = 30	1.9 [1.26, 2.51]	1.2 [0.68, 1.77]
	31–60	1.2 [0.47, 1.83]	1.0 [0.32, 1.61]
	> = 61	1.9 [0.84, 3.03]	1.2 [0.35, 2.00]
Media exposure	No	1.7 [1.18, 2.14]	1.2 [0.79, 1.66]
	Yes	2.5 [1.32, 3.70]	1.1 [0.43, 1.72]
Wealth index	Poorest	1.8 [0.77, 2.80]	1.5 [0.47, 2.43]
	Poorer	2.2 [1.04, 3.35]	1.6 [0.50, 2.64]
	Medium	0.7 [0.00, 1.39]	0.7 [0.00, 1.38]
	Richer	1.5 [0.53, 2.37]	0.7 [0.01, 1.33]
	Richest	3.5 [2.11, 4.78]	1.7 [0.84, 2.49]
Marital status	Currently in union	1.8 [1.32, 2.17]	1.2 [0.82, 1.54]
	Never/formerly in union	2.9 [0.46, 5.39]	1.5 [0.00, 3.46]
Child sex	Male	2.1 [1.42, 2.81]	1.5 [0.85, 2.13]
	Female	1.5 [0.95, 2.00]	0.9 [0.47, 1.32]
Birth weight	Large	1.3 [0.70, 1.94]	1.0 [0.45, 1.54]
	Average	1.9 [1.26, 2.62]	1.0 [0.51, 1.53]
	Small	2.3 [1.37, 3.13]	1.8 [0.97, 2.62]
Child age (months)	6–12	0.6 [0.00, 1.22]	0.6 [0.00, 1.21]
	13–23	1.5 [0.45, 2.57]	1.1 [0.14, 2.12]
	24–35	2.2 [1.12, 3.22]	1.7 [0.78, 2.63]
	36–47	2.7 [1.57, 3.83]	1.6 [0.70, 2.54]
	48–59	1.7 [0.86, 2.47]	0.8 [0.18, 1.38]
**Total**		**1.8 [1.38–2.24]**	**1.2 [0.83, 1.57]**

On the other hand, prevalence of TBM among urban residents was 1.6% (95%CI: 0.76–2.39) whereas 1.2% (95%CI: 0.76–1.56) among rural residents. Furthermore, based on maternal age the highest prevalence of TBM (1.8%, 95%CI:0.69–2.86) was observed among those in age range of 30–34 years old while the smallest prevalence (0.9%, 95%CI:0.16–1.55) in age group of 15–24 years old.

### Spatial distribution of DBM and TBM

We used Anselin Local Moran’s I [30] to detect statistically significant DBM and TBM clusters and outliers in Ethiopia. The analytic output is divided into four primary groups: two for clusters and two for outliers. High-High (HH) and Low-Low (LL) clusters highlight similar enumeration regions with high and low prevalence respectively. High-Low (HL) and Low–High (LH) identifies outliers with high prevalence surrounded by enumeration areas of low prevalence and vice versa. Plots given below show the results of cluster and outlier analysis via Anselin Local Moran’s I as well as interpolation plots for DBM and TBM.

**Fig. 2:**
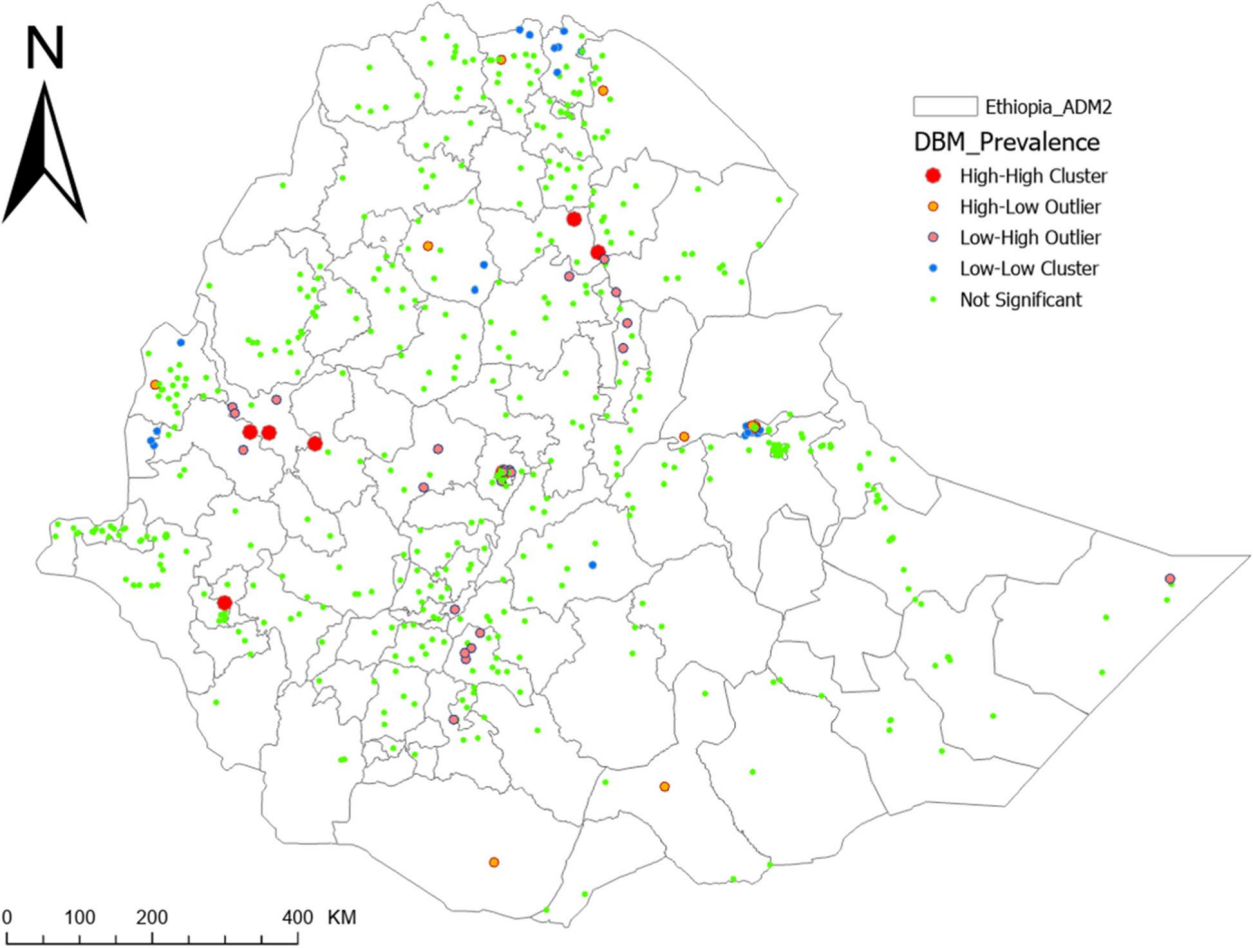
Pattern analysis (Geospatial Clustering and Hot Spot Detection) of double burden of malnutrition (DBM) among child-mother/caregiver pairs in Ethiopia

**Fig. 3:**
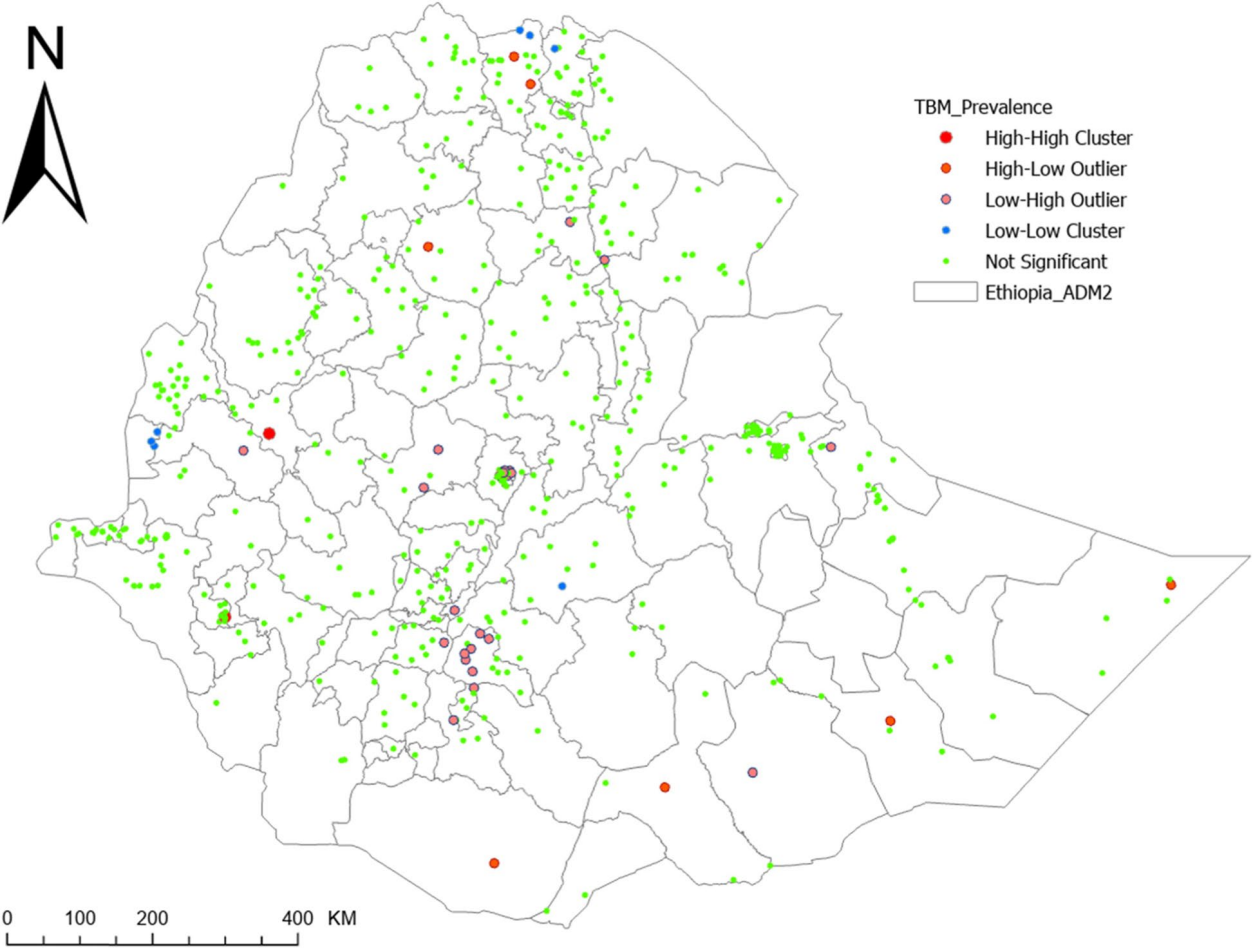
Pattern analysis (Geospatial Clustering and Hot Spot Detection) of triple burden of malnutrition (TBM) among child-mother/caregiver pairs in Ethiopia

Figure 2 depicts a cluster of DBM high values (hot spots) and low values (cold spots). This plot revealed the occurrence of hot spots of high prevalence of DBM in western and northwest Ethiopia. TBM hotspots, on the other hand, were observed in the northern Ethiopia, northwest Ethiopia, west Ethiopia, southern Ethiopia and eastern Ethiopia (Fig. 3).

**Fig. 4:**
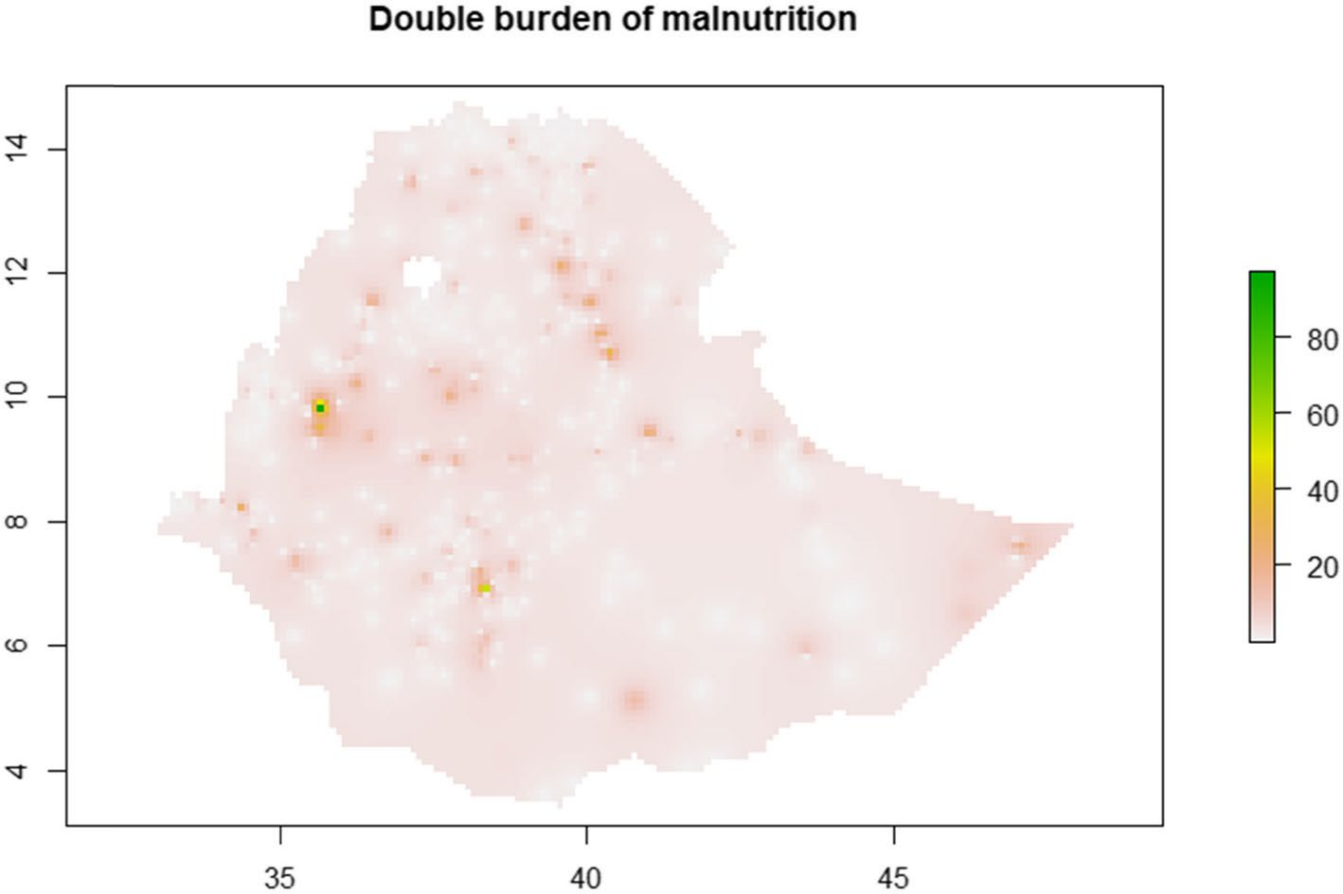
Spatial distribution for DBM via IDW interpolation

**Fig. 5:**
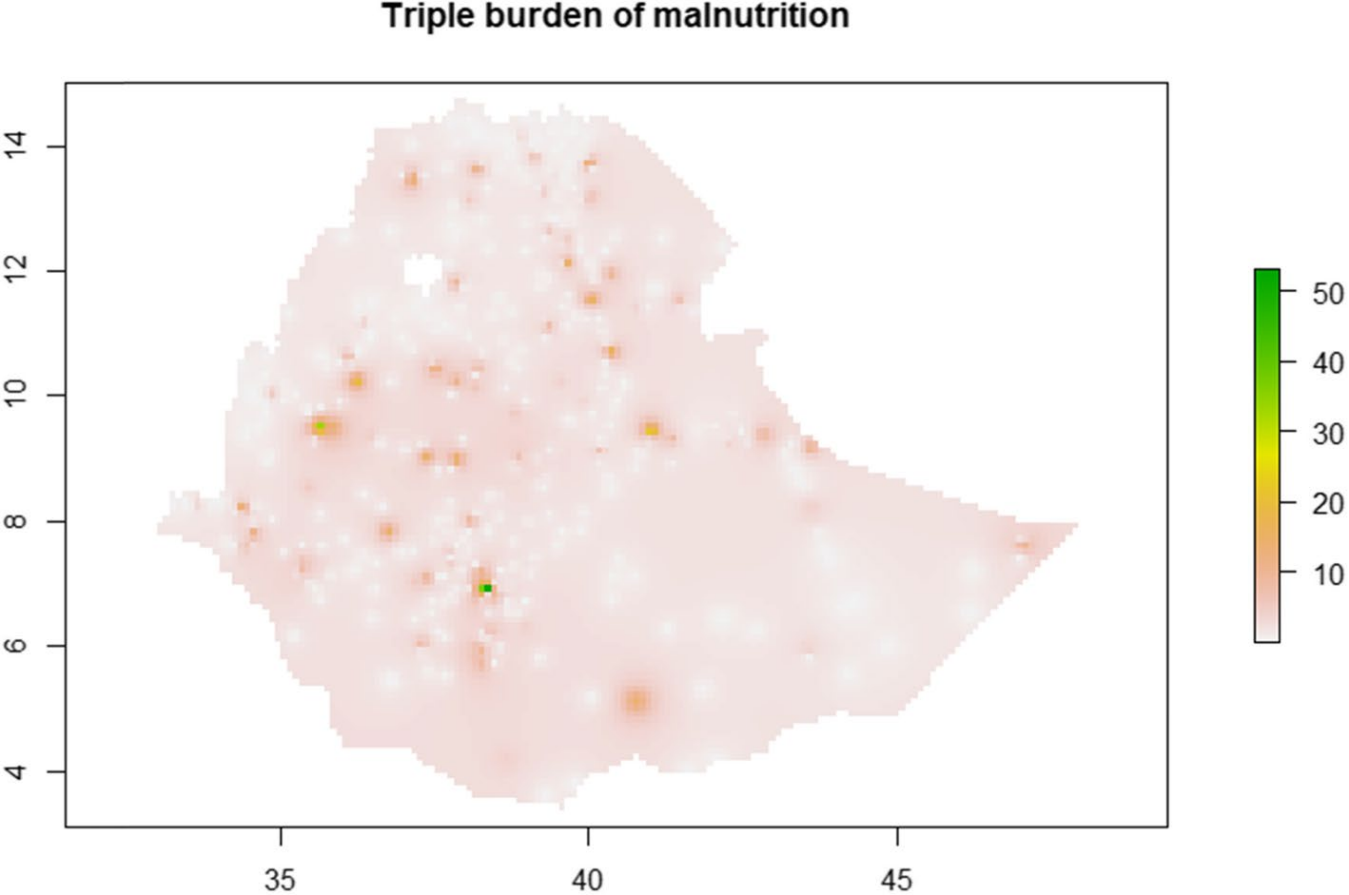
Spatial distribution for TBM via IDW interpolation

The estimated prevalence of DBM and TBM using interpolation analysis were undergone showing existence of spatial variation in prevalence of DBM and TBM, which supported by the results of cluster analysis (Fig. 4 and Fig. 5).

### Associated factors of double burden of malnutrition

The likelihood of experiencing double burden of malnutrition among child-mother/caregiver pairs are middle level wealth index as compared to the poorest [AOR = 0.23, 95%CI: 0.06, 0.89], average birth weight as compared to large birth weight [AOR = 0.26, 95%CI: 0.09, 0.80] and child age group of 24–35 months as compared to 6–12 months [AOR = 0.19, 95%CI: 0.04,0.95] (Table 4).

**Table 4:** Associated factors of DBM among child-mother pairs in Ethiopia

Variables		Coefficients	S.E	*P*-value
Toilet Facility	Open field	-0.52	0.457	0.2526
	Unimproved	0.01	0.348	0.9738
Mother’s Age	25–29	0.01	0.493	0.9775
	30–34	0.51	0.500	0.3114
	35–49	0.27	0.618	0.6583
HH size	5–7	0.01	0.450	0.9858
	8 +	0.14	0.529	0.7977
Residence	Rural	-0.26	0.507	0.6062
Education	Primary	0.57	0.564	0.3085
	No Formal	0.58	0.653	0.3774
Time To Water	< = 30 min	0.28	0.542	0.6071
	31–60 min	-0.02	0.585	0.9694
Media Exposure	Yes	0.46	0.388	0.2389
Wealth Index	Poorer	-0.39	0.549	0.4738
	Middle	-1.48	0.700	**0.0351**
	Richer	-1.08	0.635	0.0909
	Richest	-0.18	0.665	0.7868
Marital status	Never In Union/divorced/ widowed/separated	0.79	0.511	0.1230
Child sex	Female	-0.35	0.323	0.2837
Birth Weight	Average	-1.33	0.564	**0.0188**
	Small	0.07	0.337	0.8316
Child Age	13–23	1.28	0.877	0.1446
	24–35	1.62	0.799	**0.0427**
	36–47	1.33	0.779	0.0885
	48–59	1.37	0.791	0.0830

### Associated factors of triple burden of malnutrition

The likelihood of experiencing triple burden of malnutrition among child-mother/caregiver pairs are time to water source (in minutes) of <  = 30 min [AOR = 0.49, 95%CI: 0.26, 0.93] and 31–60 min [AOR = 0.41, 95%CI: 0.19, 0.89] as compared to On Premise and average birth weight as compared to large birth weight [AOR = 0.20, 95%CI: 0.05, 0.91] (Table 5).

**Table 5:** Associated factors of TBM among child-mother pairs in Ethiopia

Variables		Coefficients	S.E	*P*-value
Toilet Facility	Open field	-0.57	0.491	0.2450
	Unimproved	-0.25	0.419	0.5469
Mother’s Age	25–29	0.16	0.473	0.7328
	30–34	0.39	0.506	0.4458
	35–49	0.10	0.797	0.8959
HH size	5–7	-0.38	0.484	0.4272
	8 +	-0.34	0.607	0.5790
Educ	Primary	0.81	0.619	0.1886
	No Formal	0.85	0.733	0.2455
Distance to water source	< = 30 min	**-0.71**	**0.327**	**0.0305**
	31–60 min	**-0.89**	**0.393**	**0.0237**
	Poorer	-0.68	0.726	0.3437
Wealth Index	Middle	-1.13	0.792	0.1524
	Richer	-1.53	0.809	0.0587
	Richest	-1.31	0.788	0.0987
Child sex	Female	-0.47	0.468	0.3193
Birth Weight	Average	**-1.60**	**0.768**	**0.0375**
	Small	-0.35	0.393	0.3681
Child Age	13–23	0.92	0.961	0.3414
	24–35	1.32	0.847	0.1186
	36–47	0.64	0.847	0.4481
	48–59	0.64	0.892	0.4761
Marital	Never In Union/divorced/ widowed/separated	0.62	0.610	0.3103

## Discussion

This study identified the prevalence and spatial distributions of double burden of malnutrition and triple burden of malnutrition as well as their associated factors among child-mother pairs within the same household in Ethiopia.

In Ethiopia, the overall weighted prevalence of DBM was 1.8% (95% CI: 1.38–2.24) which is lower than 6.6% in Nepal [22]. Specifically, overweight/obese mother and stunted child were the most prevalent DBM which is in agreemen8t with a comparative study in Addis Ababa with rural district of Kersa [33] as well as in nationwide study in Nepal [18]. However, the magnitude of overweight/obese mother and stunted child, 23%, reported by Eshete et al. [18] is much greater than the result in this study. This discrepancy is because of the denominator considered. Apart from DBM, the coexistence of obese/overweight mother and anemic showed the next highest prevalence which is in agreement with similar study [18]. The overall weighted prevalence of TBM (co-existence of DBM and anemic child) was 1.2% (95%CI: 0.83–1.57) in Ethiopia which is lower than 7.0% in Nepal [22].

Though the prevalence of DBM and TBM is low, these emerging public health issues in developing countries have to be controlled so that they cannot become worse. The low prevalence of DBM and TBM in Ethiopia might be due to a lack of nutrition transition, change in activity levels, and dietary patterns resulting from a country's slow progress to higher levels of economic development [34]. Furthermore, low proportion of urban population in Ethiopia might be other reason. Similar studies conducted in various part of the world including African countries showed DBM and TBM were more likely among urban residents [34]. Hence, tailored interventions based on residence and economic status of households might help to control the prevalence of DBM and TBM among child-mother pairs in the country, Ethiopia.

Furthermore, there were identified hotspots of high prevalence of DBM as well as hotspots of high prevalence of TBM. This shows that in addition to the overall burden DBM and TBM in the country, there was spatial variation in the DBM and TBM. Accordingly, the hot spots of high prevalence of DBM were observed in western and northwest Ethiopia and hot spots of high prevalence of TBM were observed in the northern Ethiopia, northwest Ethiopia, west Ethiopia, southern Ethiopia and eastern Ethiopia. Hence, further detailed investigations on spatial attributes leading to different effects on DBM and TBM could be conducted.

Wealth status, birth weight and child age were the identified associated factors of DBM. Children from middle wealth status compared to poor wealth status (AOR = 0.23, 95% CI: 0.06–0.89) were less likely to experience DBM. Mothers from richest wealth status compared to poor wealth status (AOR = 2.46, 95% CI: 1.17–5.15) were more likely to experience DBM [18]. The occurrence of DBM could be due to the consumption of energy-dense food that leads to overweight/obesity among the mothers, while the energy dense foods are not sufficiently nutrient dense to provide the children with adequate nutrition, leading to undernutrition [8]. The DBM was more common in households with higher income [8]. As a result, more focus should be placed on delivering health information linked to the intake of energy dense foods to wealthy households.

Children with average birth weight as compared to large birth weight [AOR = 0.26, 95%CI: 0.09, 0.80] were less likely to experience DBM in agreement with a similar study [35]. Furthermore, a child with average birth weight as compared to large birth weight [AOR = 0.20, 95%CI: 0.0448, 0.909] were less likely to develop TBM. In majority of observational studies, birth weight was used as a proxy for early nutritional status. Consistent evidence suggests that higher birth weight and better nutritional status in childhood have a strong positive influence on the lean body mass in later life. Overall evidences suggest that larger birth weight in early life is associated with a higher lean body mass in later life. On the other hand, a few studies that assessed the long-term impact of protein energy supplementation in early life on the later lean body mass indicate modest and inconsistent benefit. Nutrition interventions for tackling undernutrition should aim to increase the lean body mass to address the DBM and TBM.

A child from age group of 24–35 months as compared to 6–12 months [AOR = 0.19, 95%CI: 0.04,0.95] were less likely to experience DBM. This result is in agreement with other studies [18] showing children aged between 24 and 59 months old, are more likely to encounter DBM significantly than the 0 to 23 months. A result from another study conducted among households in Myanmar and Pakistani is in agreement with this result [36]. This may be due to the combination of the different reasons; even though breast feeding has protective effect on both childhood undernutrition and maternal obesity, it is less practiced among children 24–59 months.

Likewise, results this study showed a child from a household with time to water source (in minutes) of <  = 30 min [AOR = 0.49, 95%CI: 0.26, 0.93] and 31–60 min [AOR = 0.41, 95%CI: 0.19, 0.89] as compared to on premise were less likely to develop TBM. A similar study showed that children who lived on households with access to water were found to be less likely to suffer from thinness than those who had no access [37]. Access to an improved water source is found to be an indicator of the higher probability of safe water [38]. The literature documents that the contamination of drinking water may particularly jeopardize children’s nutritional status by inhibiting their growth and health, leading to malnutrition [38].

## Limitations

The cross-sectional nature in this study, whereby it may not explain the causal and temporal relationship of associated factors with DBM and TBM. There might also be recall bias during answering child characteristics.

## Conclusion

There is low prevalence of double burden of malnutrition and triple burden of malnutrition among child-mother pairs in Ethiopia. Interventions tailored on geographic areas, wealth index, access to drinking water, birth weight and child birth could help to control double and triple burdens of malnutrition among child-mother pairs in Ethiopia.

## Data Availability

The minimal data used during the current study would be shared upon reasonable request from the correspondence.

## Abbreviations

ANC: Antenatal Care
AOR: Adjusted Odds Ratio
BMI: Body Mass Index
CI: Confidence Interval
COR: Crude Odd Ratio
CSA: Central Statistical Agency
DBM: Double Burden of Malnutrition
EDHS: Ethiopian Demographic and Health Survey
EAs: Enumeration Areas
SD: Standard Deviation
SNNPR: South Nations and Nationality of Peoples Region
SPSS: Statistical Package for Social Sciences
SSA: Sub Saharan Africa
TBM: Triple Burden of Malnutrition
USAID: United States Agency for International Development
WHO: World Health Organization
